# Adult Henoch-Schönlein purpura associated with small cell lung cancer: A case report and review of the literature

**DOI:** 10.3892/ol.2013.1274

**Published:** 2013-03-27

**Authors:** XUE-DE ZHANG, SHUAN-YING YANG, WEI LI, ZONG-JUAN MING, YAN-LI HOU, ZE-QUN NIU, YU-PING ZHANG

**Affiliations:** Department of Respiratory Medicine, The Second Affliated Hospital, Medical College of Xi’an Jiaotong University, Xi’an, Shaanxi 710004, P.R. China

**Keywords:** lung cancer, Henoch-Schönlein purpura, complications

## Abstract

The present study reports the case of a 53-year-old male who had been suffering from coughing and the presence of a blood-streaked sputum for >1 month. Chest computed tomography (CT) and a bronchoscopic brush smear were performed. The patient was subsequently diagnosed with small cell lung cancer (limited stage). The patient developed polyarthritis, abdominal pain, diarrhea and a purpuric rash at 14 days post thoracotomy surgery for lung cancer. Henoch-Schönlein purpura (HSP) was diagnosed based on the clinical symptoms. The patient received chemotherapy with steroid therapy, which resulted in complete remission of the HSP.

## Introduction

Henoch-Schönlein purpura (HSP), also known as anaphylactoid purpura, is a systemic vasculitis involving the small blood vessels with multiorgan involvement (1). The skin is a major target organ, and the hallmark of HSP is palpable cutaneous purpura; however, other organs, including the joints, gastrointestinal tract and kidneys, may be affected (1). HSP is the most common vasculitic disease of childhood, but may also occur in adults. It was first reported by Heberden in 1801 and the association between purpura and arthritis was first described by Schonlein in 1837 (2). The descriptions of gastrointestinal (GI) involvement and renal involvement were added by Henoch in 1874 and 1899 (2). Although HSP has been recognized for over 100 years (3), the exact cause of HSP is presently unknown, but malignancy may be a causative factor (4–6). The present study reports a rare case of HSP associated with small cell lung cancer. Written informed consent was obtained from the patient’s spouse for publication of this case report.

## Case report

A 53-year-old male was admitted to The Second Affiliated Hospital of Xi’an Jiatong University (Shaanxi, China) in August 2011 with symptoms of coughing and a blood-streaked sputum that had persisted for >1 month. The patient was a smoker with a 30 pack-year history of smoking. Chest computed tomography (CT) showed a mass measuring 50×40 mm in the lower lobe of the right lung. A bronchoscopy revealed an obstruction of the right B9 bronchus with a papillary tumor. A bronchoscopic brush smear was suggestive of small cell undifferentiated carcinoma. CT of the abdomen and head was negative and bone scans showed no evidence of potential metastases. Hematological investigations showed a hemoglobin level of 12.4 g/dl, a white blood cell count of 4.6×10^9^/l (normal differential) and a platelet level of 209×10^9^/l. Liver functions and blood urea nitrogen (BUN) and creatinine levels were all normal, as was the urinalysis. The final oncological diagnosis was of a limited clinical stage (T2N0M0) small cell lung cancer. Adjuvant chemotherapy consisting of cisplatin and VP-16 was initiated, followed by surgical resection. Histological studies showed small cell undifferentiated carcinoma with mediastinal lymph node metastases. The patient developed polyarthritis, abdominal pain and diarrhea, with a purpuric rash on his bilateral lower extremities and buttocks at 14 days post-surgery. The patient was not taking any medication and did not have any food allergies. Minor abdominal tenderness was revealed upon examination. A new set of hematological investigations showed a hemoglobin level of 12.9 g/dl, a white blood cell count of 8.5×10^9^/l, a platelet level of 217×10^9^/l and an erythrocyte sedimentation rate (ESR) of 28 mm/1 h. The C-reactive protein level was 52 mg/l (normal range ≤10 mg/l) and complement components C3 and C4 were measured at 94 mg/dl (normal range 85–193 mg/dl) and 17 mg/dl (normal range 12–36 mg/dl), respectively. The IgA level was recorded as 582 mg/dl (normal range 76–390 mg/dl) and the blood urea nitrogen (11.48 mmol/l), creatinine level and coagulation screen were all normal. Urinalysis revealed proteinuria of 1.52 g/24 h and hematuria of >100 red blood cells per high-power field. The patient’s blood pressure was normal. A stool examination for occult blood was positive. The tests for antistreptolysin O, rheumatoid factor (RF), anti-nuclear antibody (ANA), IgG, IgM, antineutrophil cytoplasmic antibody (ANCA) and cryoglobulin were all normal or negative. Tests for the Hepatitis B surface antigen and antibody for human immunodeficiency virus were negative. Skin and renal biopsies were not performed as the patient refused to consent to the procedures.

The patient was consequently diagnosed with HSP. A treatment was administered that consisted of 60 mg prednisolone daily and methylprednisolone pulses combined with cyclophosphamide. The skin rash, abdominal pain and polyarthritis were resolved within two weeks. The patient also received post-operative thoracic radiotherapy (50 Gy in 25 sessions). Chemotherapy was deferred due to a concern for the increased likelihood of BUN and proteinuria associated with the treatment. The proteinuria decreased to 668.25 mg/24 h and the hematuria improved 2 months after the start of steroid treatment. Chest CT revealed a left lung metastasis from the primary carcinoma, therefore, chemotherapy consisting of carboplatin and VP-16 was administered immediately. The proteinuria improved to 147.6 mg/24 h subsequent to one cycle of chemotherapy. The patient continued chemotherapy for a total of 4 cycles until the hematuria had completely resolved and the proteinuria was normal. A liver metastasis was identified by abdominal CT 6 weeks after the 4 cycles of chemotherapy. The patient received interventional therapy for the liver metastasis. The patient’s general condition gradually deteriorated and he succumbed one year and three months subsequent to the diagnosis of small cell lung cancer.

## Discussion

HSP is a multisystem IgA-mediated vasculitis with a self-limited course, which may affect the skin, joints, gastrointestinal tract and kidneys. A likely mechanism behind this involves a process by which the complexes of immunoglobulin A (IgA) and complement component 3 (C3) become deposited on arterioles, capillaries and venules, thus increasing their brittleness and permeability. This is followed by deposits on the skin, joints, gastrointestinal tract, kidneys and other organs, thus causing bleeding and other symptoms. Purpura, arthritis and abdominal pain are known as the ‘classic triad’ of HSP. HSP occurs more often in children than in adults. The outcome of HSP predominantly depends on the degree of renal involvement.

The diagnosis of HSP is based on the combination of the symptoms, as few other diseases cause the same symptoms together. The diagnostic criteria for HSP, according to the American College of Rheumatology, are palpable purpura, bowel angina, a patient age of ≤20 years and histological changes of leukocytoclastic vasculitis. A diagnosis of HSP is 87.1% sensitive and 87.7% specific when two or more of these criteria are present (7). The present study patient had palpable purpura of the lower extremities and buttocks combined with bowel angina, thereby meeting the American College of Rheumatology criteria for the diagnosis of HSP. Additionally, the patient had arthritis and hematuria and an increased serum IgA, which provided further support for the diagnosis of HSP.

The exact etiology of HSP is unknown, but factors including bacterial infection or drugs, foods, allergens (8) and autoimmune connective tissue disease (9,10) have been documented. In the literature, associations between solid organ malignancies and HSP have been reported in cases when there were no known triggering factors (11). Several studies have reported the correlation between HSP and malignancies, including esophageal, lung, breast, gastric and prostate cancer, and hematological malignancies (11–13). Solid tumors are more common than hematological malignancies in association with HSP (15). Lung cancer is the most common solid malignancy associated with HSP. In the study by Zurada *et al*(15), 3l patients with HSP had underlying malignancies, among which solid tumors accounted for 61% and lung cancer accounted for 25%. To the best of our knowledge, 13 cases of HSP associated with lung cancer have been reported to date (Table I) (16–27). In these 13 cases there were 12 male patients and one female patient with an average age of 65.7 years (50–79 years). These cases included 8 diagnoses of squamous cell carcinoma, 3 of adenocarcinoma and 2 of small cell lung cancer. In 6 cases, the onset of the HSP and lung cancer processes was synchronous. In 6 cases, HSP preceded lung cancer and in 1 case HSP occurred after lung cancer had been diagnosed. All 13 patients exhibited skin and kidney involvement, 7 patients had gastrointestinal symptoms and 9 patients had joint involvement. These studies aided in the confirmation of the correlation between lung cancer and HSP. The patient in the present study had no correlation with these pathogenic factors. The onset of HSP occurred 14 days after surgical resection, making it likely that the HSP was associated with the lung cancer.

In the 13 literature cases, the treatment for HSP ranged from oral steroids to a combination of intravenous methylprednisolone followed by prednisone. Other possible regimens include steroids/azathioprine, and intravenous immunoglobulin (IVIG). Although an explicit treatment for HSP associated with lung cancer has not been identified, several studies have presented a treatment for HSP associated with lung cancer. The lung cancer therapies induced a complete remission of HSP in 4 cases (16,17,22,13) while steroid hormones were required for an improvement in HSP in another 6 patients (18,21,24–27). Only one case (18) reported a recurrence of HSP. The patient in the present study initially received prednisolone and methylprednisolone pulses combined with cyclophosphamide. This treatment achieved a partial remission of the HSP with the proteinuria decreased to 668.25 mg/24 h. Following this, a total of 4 cycles of chemotherapy were performed to treat the left lung metastasis. Complete remission of the HSP was achieved, suggesting that the treatment for malignancy may contribute to a complete remission of HSP.

The exact correlation between HSP and malignancies remains somewhat unclear. It is possible that malignancies and HSP in patients may be correlated with the generation and expression of tumor-associated antigens. These antigens, as heterologous material, are able to stimulate the body to produce aberrant antibodies. These antibodies react to antigens and lead to the resultant immune complex deposition within vessel walls. Moreover, tumor-associated antigens reduce the clearance of circulating immune complexes (28,29). In addition, tumor-associated antigens may cause dysfunctionality in lymphocytes and lead to the immunoglobulin subtypes shifting from IgM to IgA,. Once this happens, a large number of inflammatory cytokines may be released and vascular inflammation may eventually occur (30). The treatment of malignant tumors may also cause the occurrence of HSP. It is possible that radiotherapy, chemotherapy and surgical resection change the surface antigens of tumor cells or that tumor-associated antigens are released within the tumor cells following their destruction (31). It has previously been demonstrated that numerous chemotherapeutic agents, including erlotinib (32), cytarabine (33) and anastrozole (34), result in HSP.

The patient in the present study developed HSP following surgical resection, however, the exact nature of the correlation between HSP and lung cancer in this case remains poorly defined. The patient did not receive any medication and no known food allergens were consumed. It was therefore postulated that the occurrence of HSP in this patient may have been attributed to the malignancy itself. We suggest that the tumor-associated antigen may have changed or that there may have been internal antigen exposure to the blood circulation during surgical resection, thus leading to the occurrence of HSP.

The present study presents a rare case of HSP associated with small cell lung cancer. To the best of our knowledge, this case is only the third patient with HSP presenting with small cell lung cancer. In addition, this appears to be the only case of HSP occurring post-surgery. Although the present study only involved 1 case, a review of the literature also aided in the confirmation of a correlation between lung cancer and HSP. The present study also suggested that the tumor-associated antigen may change in the treatment process of cancer and lead to the occurrence of HSP. The specific mechanisms by which this occurs will require further research in the future. It remains unclear which is the most efficient treatment for HSP associated with malignant tumors, and since corticosteroid therapy may not cause complete remission, future anti-tumor therapies may play a more crucial role in the treatment of such patients.

## Figures and Tables

**Table I:** Characteristics of patients with Henoch-Schönlein purpura (HSP) associated with lung cancer.

First author (Ref.)	Age (years)/gender	Histology	Onset of HSP in relation to lung cancer	Organ involvement	Treatment for HSP	Response to treatment	Treatment for lung cancer
Cairns (16)	63/M	Squamous	8 months prior	Skin, renal, joint	Not performed	Remission	Not performed
	73/M	Squamous	Synchronous^a^	Skin, renal, joint, GI tract	Not performed	Remission	Surgery
Maurice (17)	59/M	Squamous	Synchronous	Skin, renal, joint	Not performed	Remission	Surgery
Mitchell (18)	57/M	Squamous	22 months prior	Skin, renal, GI tract	Prednisone and azathioprine	Remission	Surgery
Pfitzenmeye (19)	79/M	Squamous	6 months prior	Skin, renal	Not performed	Not mentioned	Not performed

Gutierrez (20)	78/M	Squamous	Synchronous	Skin, renal, GI tract, joint	Not available	Not mentioned	Not available
Frigui (21)	50/M	Squamous	6 months prior	Skin, renal, GI tract, joint	Methylprednisolone and prednisone	Remission	Chemotherapy, irradiation
Blanco (22)	67/M	Small cell	Synchronous	Skin, renal	Not performed	Remission	Chemotherapy
Ponge (23)	75/M	Small cell	8 months prior	Skin, renal, joint	Not performed	Remission	Chemotherapy
Weiler Bisig (24)	63/M	Adenocarcinoma	Synchronous	Skin, renal,	Prednisone GI tract	Remission	Chemotherapy
Solans Laque (25)	58/M	Adenocarcinoma	Synchronous	Skin, renal, GI tract, joint	Prednisone and γ globulins	Remission	Surgery
Mifune (26)	61/M	Adenocarcinoma	Synchronous	Skin, renal, GI tract, joint	Prednisone	Remission	Chemotherapy
Podjasek (27)	71/F	Squamous	3 months after	Skin, joint	Prednisone	Remission	Surgery, chemotherapy, irradiation

a Occurred within 1 month of each other. M, male; F, female; GI, gastrointestinal.
